# Research integrity within systematic reviews: investigating the prevalence of studies by authors with multiple retraction histories in Cochrane reviews

**DOI:** 10.1186/s41073-026-00205-2

**Published:** 2026-06-22

**Authors:** Theint Moh Moh Zaw, James Heathers, Gideon Meyerowitz-Katz

**Affiliations:** 1 The School of Public Health, University of Sydney, Sydney, NSW 2006 Australia; 2 Medical Evidence Project, Center for Scientific Integrity, New York, NY 10018 USA; 3 Linnaeus University, Vaxjo, 35195 Sweden; 4 Epidemiologist | Western Sydney Diabetes, Sydney, NSW Australia; 5 School of Nursing and Midwifery, University of Wollongong, Wollongong, NSW 2522 Australia

**Keywords:** Research integrity, Research misconduct, Fraudulent studies, Authors with multiple retraction hitories, Cochrane reviews, Systematic review reliability, Sensitivity analysis

## Abstract

**Background:**

Research misconduct poses a significant threat to the integrity of evidence synthesis. Although Cochrane reviews are known for rigorous methodological standards, the potential inclusion of studies authored by researchers with substantial retractions records raises concerns about the reliability of their conclusions.

**Objectives:**

This study aims to (a) quantify the extent to which Cochrane reviews include or cite papers authored by researchers with at least 24 retractions (as listed in the Retraction Watch Leaderboard),and (b) examine whether sensitivity analyses were performed and identify the stated reasons for excluding those studies from the reviews.

**Methods:**

The Retraction Watch database was used to identify researchers with 24 or more retractions in the database. This was done with custom code in Stata, created to identify individuals with > 24 entries. Data on Cochrane’s systematic reviews that included or referenced the studies by authors with 24 or more retraction records was collected. For reviews where these studies were excluded, the stated reasons for exclusion were recorded. We also assessed the frequency and nature of sensitivity analyses conducted to account for potential bias from problematic studies.

**Results:**

Of 9,323 Cochrane Reviews, 81(0.9%)included or cited at least one potentially fraudulent study. 57(0.6%) of these reviews referenced and 32(0.3%) included such studies in meta-analyses. In cases where such studies were excluded, the justification was typically unrelated to trustworthiness. Of the 32 reviews that include potentially fraudulent studies, only a minority of reviews (19%, 6 out of 32) conducted sensitivity analyses after paper exclusions, primarily due to retracted papers, with only one analysis conducted in response to potential fraudulent studies.

**Conclusion:**

The inclusion or insufficient handling of studies by highly retracted researchers in Cochrane reviews without sensitivity analysis highlights the concerns regarding the integrity and reliability of evidence used to guide clinical practice. This study underscores the need for formal mechanisms to assess author trustworthiness, recommends transparency in handling these studies, and calls for sensitivity analyses to be mandatory.

## Introduction

Historically, two-thirds of scientific retractions are due to scientific misconduct, with studies reporting rates of 67.4% [1] and 65.3% [2]. The present proportion is undefined but almost certainly higher, given that recent total retraction figures are dominated by the more than 10,000 papers being systematically retracted from the Hindawi journal group for various forms of misconduct (fabrication, manipulated peer review, etc.), and that this is the approximate total of all retractions from 2020-2022 [3]. This can be seen as part of a broader increase over time – retraction due to fraud increased more than sevenfold between 2004 and 2009 [4]. And while the total number of retracted papers is rising each year, the percentage of retracted papers is also rising (from about 0.05% in 2007, to 4 times that in 2022) [5].

Given the increase in volume and percentage, it feels reasonable that the number of retracted papers being cited will also rise over time [6]. Likewise, and crucially here, the pool of ‘untrustworthy’ papers (those which contain an untrustworthy author i.e one with several other already retracted papers with a similar involvement as an author) will also increase.

At present, we can find no record of a systematic investigation into the broader work of authors with multiple retraction histories, and no formal recommendation or checklist, such as the Cochrane Risk of Bias tool, exists for conducting a review that includes a scientometric analysis of the cited authors. Given the unreliability of such studies, it is clear that they should be analyzed at a minimum or excluded entirely from systematic reviews. However, at present, there is no evidence that this is routinely done.{Bakker, 2023 #489} [7] Rather, Steen et al. (2013) suggest that retracted papers in medical studies continue to be cited as if they had not been retracted [8]. Previous meta-epidemiological work found that a significant number of systematic reviews and clinical guidelines include already retracted randomized controlled trials (RCTs), and only a small percentage of these publications corrected their findings after the retraction of the included RCTs was announced [9].

The urgency of all of the above is increased if untrustworthy papers and the papers that cite them are medical or pharmacological. These disciplines have a unique ability to directly and sometimes immediately threaten human health as they can change medical practice [10]. At the same time, medical papers (a) show a higher incidence rate; Martinson and colleagues found that medical researchers reported misconduct at higher rates than their peers in other disciplines, including falsification and fabrication of data [11], and (b) medical researchers dominate the Retraction Watch Leaderboard – to date, 6 out of the 10 authors with multiple retraction records in history were medical researchers. Such authors have historically dominated retraction statistics [12]. Finally, several cases exist where highly retracted authors have only had part of their known body of unreliable work retracted.

The harm of this has been previously quantified: from 2000-2010, medical retractions directly affected at least 9,181 patients, and secondary studies that cited these papers influenced the treatment of over 70,500 patients [10]. Likewise, several serious individual cases have been identified. The Poehlman case exemplifies how research misconduct can directly misguide clinical practice; his falsified data on hormone replacement therapy risked promoting harmful treatments for postmenopausal women, potentially exposing patients to serious adverse medical outcomes such as cardiovascular disease and cancer [13]. Although many of his studies were eventually retracted, the case highlights that retractions alone may not fully mitigate harm, as misleading findings can persist in the literature and continue to influence clinical decisions.

Here, we seek to quantify how many Cochrane reviews include or cite these papers, and determine how they might subsequently affect treatment recommendations and clinical decision-making. Cochrane reviews set a de facto gold standard for medical reviews, clinical guidelines and policy making. The reviewers who produce them have access to tools to detect potentially fraudulent studies, but these tools may not always be used and, considering the evolving landscape of scientific malpractice, may be insufficient to detect potentially citable but problematic papers [14]. Cochrane reviews significantly impact medical practices and healthcare decisions, making it vital to ensure their integrity [14].

In May 2021, entry into the Retraction Watch Leaderboard _an unofficial but frequently updated public list of authors with multiple retraction histories_ required a minimum of 24retracted publications [15]. In this study, this threshold was used as a pragmatic and objective criterion to define authors with multiple retraction records. The number reflects the toal retracted articles attributed to an author in the Retraction Watch Database at he time of data extraction, rather than a predefined or universally accepted cutoff. Studies authored by researchers with multiple retraction histories may be considered potentially fraudulent and elevate the risk of compromised integrity. This work seeks to (a) quantify how many Cochrane reviews include or cite papers by researchers with multiple retraction histories, and (b) determine the presence of sensitivity analyses and the exclusion criteria used in reviews if those papers are excluded from the reviews.

## Methods

This study employed a retrospective, descriptive analysis using a systematic review methodology. This method allowed for an extensive assessment of Cochrane reviews to identify those that include or reference studies authored by researchers with multiple retraction histories.

### Data sources

The primary data source for this research was the “Cochrane Database of Systematic Reviews (CDSR) [16],” which contains 9323 Cochrane reviews when searches were conducted in 2024. In addition, the Retraction Watch database [15] was used to identify authors with 24 or more entries in the databases. This number reflects the total number of retracted studies identified within the included reviews based on the Retraction Watch Database at the time of data extraction, rather than a predefined or universal threshold. We used entries into the databases as a proxy for retractions. In practice, this expands the definition of "highly retracted" to include those with several retractions and a number of corrections to their papers. We used a custom script to Stata to identify authors with 24 or more entries into the Retraction Watch database.

### Searching data

Using the Retraction Watch database [15], authors who have had 24 or more entries in the dataset for retraction or correction of their academic work were identified. The search of authors with multiple retraction records identified a total of 281 names with > 24 entries into the Retraction Watch databases at this time. Then, we used the advanced search tools provided by the Cochrane database of systematic reviews to search for systematic reviews that reference or include studies by these authors. Author names were entered in the “All Text” option in the Cochrane advanced search tool to perform a comprehensive search across all sections of the reviews, as citations occur across multiple sections of the papers. When full first and last name combination did not yield results, the last name followed by the initial letter of the first name was used (i.e. “Asemi Z” for “Zotellah Asemi”). For authors with common last names, particularly Chinese and Korean authors, semicolons were used to distinguish between individuals.

### Data collection and categorization

For each review, data were extracted on the authorship, title, topic area, and publication data of studies by authors with multiple retraction histories. After retrieval, the reference section was examined to determine if studies were (a) included in the review, (b) excluded from the review, or (c) cited elsewhere but not considered for the review. Data were initially collected by one author and subsequently verified by a second author to ensure accuracy and disagreements were resolved through discussion. Any missing or inconsistent data, particularly regarding author names, were manually addressed and reviewed. Similar author names were cross-checked to avoid misattribution.

### Data analysis

If the studies authored by 281 researchers (with > 24 entries into the Retraction Watch databases) were included in the reviews, any evidence of a sensitivity or subgroup analysis was recorded. If studies were excluded from the reviews, the reasons for their exclusion were recorded (i.e. excluded due to methodological flaws, the authors’ credibility, etc.). Data extraction was performed by one author and verified by a second author, with disagreements resolved through discussion to ensure accuracy and consistency. Additionally, we assessed whether the retraction history of any cited authors with multiple retraction records was explicitly knowledged or discussed.

### Outcomes measurement

Three primary outcomes were assessed:

1. The number of Cochrane systematic reviews that include or reference studies by authors with 24 or more retractions
2. The number of Cochrane systematic reviews that exclude studies from authors with 24 or more retractions in their meta-analyses and their stated reasons
3. The number of Cochrane systematic reviews that have done sensitivity analyses and their nature

## Results

**Fig. 1:**
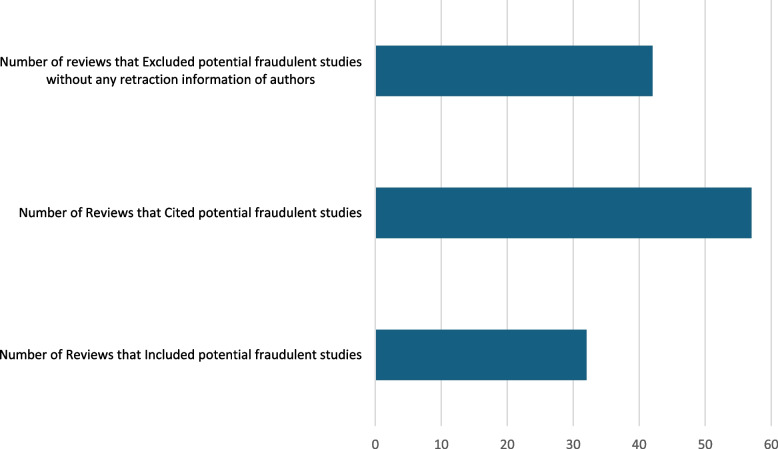
Number of reviews that excluded, cited or included potential fraudulent studies

Of 9,323 Cochrane Reviews, 57 systematic reviews (0.6%, 57 out of 9,323) that reference 78 total studies by authors with multiple retraction records were identified (Table 1), indicating that they were cited but not included in the review’s analyzed data (Table 1, Fig. 1) [17]. 32 systematic reviews (0.3%, 32 out of 9,323) included at least one study of 53 studies from authors with multiple retraction records in their meta-analyses (Table 2, Fig. 1). In 8 reviews (0.1%, 8 out of 9,323), such papers are both included and referenced. Therefore, a total of 81 (0.9%, 81 out of 9,323) Cochrane reviews have at least one study of authors with multiple retraction histories included or cited. In 42 reviews (0.5%, 42 out of 9,323), studies from authors with multiple retraction records were excluded due to the reason for not meeting the specified inclusion criteria of the reviews, rather than due to retraction notices associated with the authors (Fig. 1).

**Table 1:** Systematic reviews with the number of studies, by authors with multiple retraction records, referenced

Authors with 24 or More Retractions (Number of Retractions)	Systematic Review Title	Field of Medicine	Date of Publication	Number of Studies, by authors with multiple retraction records, Referenced
Joachim Boldt (220)	Platelet-rich-plasmapheresis for minimising peri-operative allogeneic blood transfusion	Hematology	16/03/2011	2
	Colloid solutions for fluid resuscitation	Intensive care medicine	11/07/2012	2
	Human albumin solution for resuscitation and volume expansion in critically ill patients	Intensive care medicine	09/11/2011	1
	Anti-frinolytic use for minimising perioperative allogeneic blood transfusion	Hematology	16/03/2011	1
	Non-pharmacological interventions for chronic pain in multiple sclerosis	Pediatrics	19/12/2018	1
	Pentoxifylline for treatment of sepsis and necrotizing enterocolitis in neonates	Infection	20/06/2023	1
	Whole body vibration exercise training for fibromyalgia	Rheumatology	26/09/2017	2
	Probiotic treatment for women with gestational diabetes to improve maternal and infant health and well-being	Obstetrics	24/06/2020	4
	Treatments for women with gestational diabetes mellitus: an overview of Cochrane systematic reviews	Obstetrics	14/08/2018	3
Ahmed Badawy(33)	Endometrial injury for pregnancy following sexual intercourse or intrauterine insemination	Obstetrics	24/10/2022	3
Annarosa Leri(48)	Stem cell treatment for acute myocardial infection	Cardiology	07/08/2024	1
Bin Liu (33)	Xpert MTB/RIF Ultra and Xpert MTB/RIF assays for extrapulmonary tuberculosis and rifampicin resistance in adults	Infectious diseases	15/01/2021	1
Bo Liu (28)	Covid 19 and its cardiovascular effects: a systematic review of prevalence studies	COVID-19	11/03/2021	1
Chao Zhang (25)	Covid 19 and its cardiovascular effects: a systematic review of prevalence studies	COVID-19	11/03/2021	1
Dan Liu (32)	Covid 19 and its cardiovascular effects: a systematic review of prevalence studies	COVID-19	11/03/2021	1
Fang Liu (28)	Covid 19 and its cardiovascular effects: a systematic review of prevalence studies	COVID-19	11/03/2021	1
Fang Wang (29)	Selenium for preventing cancer	Oncology	29/01/2018	2
	Anti-vascular Endothelial growth factor biosimilars for neovascular age-related macular degeneration	Ophthalmology	03/06/2024	1
Feng Wang (48)	Menopausal status, ultrasound biomarker tests in combination for the diagnosis of ovarian cancer in symptomatic women	Gynecology	26/07/2022	1
Hao Wang (34)	Covid 19 and its cardiovascular effects: a systematic review of prevalence studies	COVID-19	11/03/2021	1
Hong Zhang (50)	Tamoxifen for adults with hepatocellular carcinoma	Hepatology	12/08/2024	1
Hui Wang (78)	Covid 19 and its cardiovascular effects: a systematic review of prevalence studies	COVID-19	11/03/2021	2
Jean Christophe Lagier (53)	Covid 19 and its cardiovascular effects: a systematic review of prevalence studies	COVID-19	11/03/2021	1
Jia Liu (32)	Covid 19 and its cardiovascular effects: a systematic review of prevalence studies	COVID-19	11/03/2021	2
Jian Wang (50)	Radix Sophorae flavescent is versus other drugs or herbs for chronic hepatitis B	Hepatology	24/06/2019	1
Jie Liu (43)	Covid 19 and its cardiovascular effects: a systematic review of prevalence studies	COVID-19	11/03/2021	1
Jie Yang (27)	Covid 19 and its cardiovascular effects: a systematic review of prevalence studies	COVID-19	11/03/2021	1
Jing Yang (38)	Chinese herbal medicine for oesophageal cancer	Oncology	22/01/2016	1
Jun Li (71)	Perioperative enhanced recovery programmes for women with gynecological cancers	Gynecology	15/03/2022	2
Kai Liu (30)	Covid 19 and its cardiovascular effects: a systematic review of prevalence studies	COVID-19	11/03/2021	1
Lei Chen (31)	Covid 19 and its cardiovascular effects: a systematic review of prevalence studies	COVID-19	11/03/2021	1
Li Liu (40)	Covid 19 and its cardiovascular effects: a systematic review of prevalence studies	COVID-19	11/03/2021	1
Lin Zhang (43)	Covid 19 and its cardiovascular effects: a systematic review of prevalence studies	COVID-19	11/03/2021	1
Meng Zhang (28)	Covid 19 and its cardiovascular effects: a systematic review of prevalence studies	COVID-19	11/03/2021	1
Min Wang (37)	Diabetes as a risk factor for tuberculosis disease	Endocrinology	23/08/2024	1
Ming Li (51)	Covid 19 and its cardiovascular effects: a systematic review of prevalence studies	COVID-19	11/03/2021	1
Nan Li (48)	Undernutrition as a risk factor for tuberculosis disease	Nutrition	11/06/2024	2
Ping Wang (34)	Oral Hygiene care for critically ill patients to prevent ventilator-associated pneumonia	Infection	24/12/2020	2
Sanjeev Banerjee (37)	Short acting insulin analogues versus regular human insulin for adults with type 1 diabetes mellitus	Endocrinology	30/06/2016	1
Tao Li (42)	Covid 19 and its cardiovascular effects: a systematic review of prevalence studies	COVID-19	11/03/2021	1
Tao Liu (61)	Covid 19 and its cardiovascular effects: a systematic review of prevalence studies	COVID-19	11/03/2021	3
	Diabetes as a risk factor for tuberculosis disease	Endocrinology	23/08/2024	1
Wei Li (94)	Covid 19 and its cardiovascular effects: a systematic review of prevalence studies	COVID-19	11/03/2021	1
Wei Liu (77)	Covid 19 and its cardiovascular effects: a systematic review of prevalence studies	COVID-19	11/03/2021	2
	Cognitive behavioural therapy for chronic fatigue syndrome in adults	Neurology	16/07/2008	1
	Measures implemented in the school setting to contain the Covid-19 pandemic	COVID-19	02/05/2024	1
Wei Zhou (26)	Percutaneous vertebroplasty for osteoporotic vertebral compression fracture	Orthopedics	06/11/2018	1
Xiang Li (37)	Antivascular endothelial growth factor biosimilars for neovascular age-related macular degeneration	Ophthalmology	03/06/2024	1
Yan Li (74)	Chinese herbal medicines for subfertile women with polycystic ovarian syndrome	Gynecology	04/06/2021	1
Yan Liu (53)	Covid 19 and its cardiovascular effects: a systematic review of prevalence studies	COVID-19	11/03/2021	1
Yan Yang (28)	Covid 19 and its cardiovascular effects: a systematic review of prevalence studies	COVID-19	11/03/2021	2
	Acupuncture for acute hordeolum	Traditional medicine	09/02/2017	1
Yi Zhang (44)	Covid 19 and its cardiovascular effects: a systematic review of prevalence studies	COVID-19	11/03/2021	1
Ying Liu (62)	Covid 19 and its cardiovascular effects: a systematic review of prevalence studies	COVID-19	11/03/2021	1
Ying Wang (60)	Herbal medicines for fatty liver diseases	Hepatology	24/06/2013	1
	Physical interventions to interrupt or reduce the spread of respiratory viruses	Infectious diseases	30/01/2023	2
Yu Zhang (62)	Diabetes as a risk factor for tuberculosis disease	Endocrinology	23/08/2024	1

**Table 2:** Systematic reviews with the number of studies, by authors with multiple retraction histories, included

Authors with 24 or More Retractions (Number of Retractions)	Systematic Review Title	Field of Medicine	Date of Publication	Number of Studies, by authors with multiple retraction histories, Included
Joachim Boldt (220)	Platelet-rich-plasmapheresis for minimising peri-operative allogeneic blood transfusion	Hematology	16/03/2011	4
	Human albumin solution for resuscitation and volume expansion in critically ill patients	Intensive care medicine	09/11/2011	1
	Anti-frinolytic use for minimising perioperative allogeneic blood transfusion	Hematology	16/03/2011	3
	Patient controlled opioid analgesia versus non-patient controlled opioid analgesia for postoperative pain	Intensive care medicine	02/06/2015	1
Yoshihiro Sato (122)	Interventions for preventing falls in older people living in the community	Orthopedics	12/09/2012	1
Jun Iwamoto (90)	Etidronate for the primary and secondary prevention of osteoporotic fracture sin postmenopausal women	Orthopedics	09/04/2024	3
	Interventions for preventing falls in older people living in the community	Orthopedics	12/09/2012	2
	Exercise for improving balance in older people	Orthopedics	09/11/2011	1
Zatollah Asemi(71)	Different types of dietary advice for women with gestational diabetes mellitus	Obstetrics	25/02/2017	4
	Calcium supplementation for people with overweight or obesity	Nutrition	09/05/2024	2
	Calcium supplementation during pregnancy for preventing hypertensive disorders and related problems	Obstetrics	01/10/2018	3
	Antioxidants for adults with chronic kidney disease	Nephrology	02/11/2023	2
	Probiotic treatment for women with gestational diabetes to improve maternal and infant health and well-being	Obstetrics	24/06/2020	5
Sherief Abd-Elsalam(8)	Chloroquine or hydroxychloroquine for prevention and treatment of COVID-19	COVID-19	02/12/2021	1
	Platelet count, spleen length and platelet count-to-spleen length ratio for the diagnosis of oesophageal varices in people with chronic liver disease or portal vein thrombosis	Hepatology	26/04/2017	3
	Interventions for hirsutism (excluding laser and photoepilation therapy alone)	Endocrinology	28/04/2015	2
	Chinese herbal medicine for treating osteoporosis	Orthopedics	06/03/2014	1
Jian Wang (50)	Radix Sophorae flavescent is versus other drugs or herbs for chronic hepatitis B	Hepatology	24/06/2019	1
Kei Satoh (38)	Interventions for preventing falls in older people living in the community	Orthopedics	12/09/2012	2
Lei Zhang (83)	Menopausal status, ultrasound biomarker tests in combination for the diagnosis of ovarian cancer in symptomatic women	Gynecology	26/07/2022	1
Wei Xu (26)	Chinese herbal medicine for treating osteoporosis	Orthopedics	06/03/2014	1
Yan Chen (47)	Menopausal status, ultrasound and biomarker tests in combination for the diagnosis of ovarian cancer in symptomatic women	Gynecology	26/07/2022	1
Yan Li (74)	Chinese herbal medicines for subfertile women with polycystic ovarian syndrome	Gynecology	04/06/2021	1
	Prognosis of adults and children following a first unprovoked seizure	Neurology	23/01/2023	1
	Xpert MTB/RIF Ultra and Xpert MTB/RIF assays for extrapulmonary tuberculosis and rifampicin resistance in adults	Infectious diseases	15/01/2021	1
Yan Wang (69)	Acupuncture for acute hordeolum	Traditional medicine	09/02/2017	1
Yi Wang (39)	Treatment of periodontitis for glycemic control in people with diabetes mellitus	Endocrinology	14/04/2022	1
Ying Wang (60)	Herbal medicines for fatty liver diseases	Hepatology	24/06/2013	1
	Menopausal status, ultrasound and biomarker tests in combination for the diagnosis of ovarian cancer in symptomatic women	Gynecology	26/07/2022	1
Zi Feng Zhang (35)	Chinese herbal medicines for treating osteoporosis	Orthopedics	03/06/2014	1

The medical fields represented in these reviews were diverse (Table 3), including COVID-19, Orthopedics, Endocrinology, Obstetrics and Gynecology, Hepatology, Infectious diseases, Nutrition, Intensive care management, Neurology, Ophthalmology, Oncology, Hematology, Infection, Nephrology, Chinese traditional medicine, Cardiology, Rheumatology and Pediatrics. Among these, COVID-19 emerged as the most frequent topic, accounting for 26 reviews with 31 cited studies (Table 3), followed by Orthopedics (9 reviews including 11 studies in their meta-analyses).

**Table 3:** Systematic reviews with the number of studies, by authors with multiple retraction histories, included or cited in the reviews according to the field of medicine

Field of Medicine	Cochrane Reviews that cite or include studies by authors with multiple retraction histories	Studies, by authors with multiple retraction histories included in Reviews	Studies, by authors with multiple retraction histories Cited in Reviews
COVID-19	26	1	31
Orthopedics	9	11	1
Endocrinology	6	3	4
Obstetrics	6	13	10
Gynecology	4	4	4
Hepatology	4	5	3
Infectious diseases	3	1	3
Intensive care medicine	3	2	3
Neurology	2	1	1
Ophthalmology	2	0	2
Oncology	2	0	3
Hematology	2	7	3
Infection	2	0	3
Nephrology	2	2	0
Traditional medicine	2	1	1
Cardiology	1	0	1
Rheumatology	1	0	2
Nutrition	1	2	2
Pediatrics	1	0	1

Other prominent fields were endocrinology (6 reviews including 3 studies with 4 otherwise cited), obstetrics (5 reviews including 13 studies with 10 otherwise cited (Table 3). Gynecology, Hepatology, Hematology, Infectious diseases and Intensive care medicine showed a moderate level of engagement (e.g. hepatology; 4 reviews including 5 studies with 3 otherwise cited. Cardiology, rheumatology, pediatrics, nephrology, oncology and ophthalmology showed minimal interaction (1 or 2 studies only; Table 3).

**Fig. 2:**
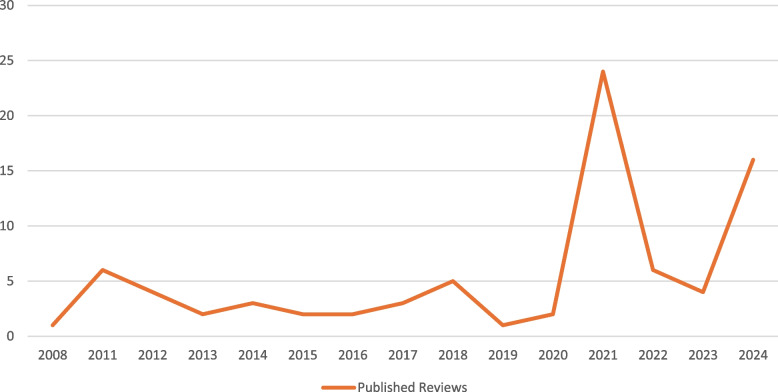
Yearly trend of published cochrane reviews that included or cited potential fraudulent studies

Among 81 reviews that included or cited studies of the authors with multiple retraction records from 2008 to 2024, 64% (52 reviews) were published after the year 2020 (Fig. 2). The year 2021 recorded the highest number of reviews published with 30% (24 reviews), while the year 2024 followed the second highest, recording 20% (16 reviews) published that included or cited such problematic studies (Fig. 2).

Sensitivity analyses were conducted in only 19% (6 out of the 32) of reviews which explicitly included the results of studies by authors with multiple retraction histories. Of these, only one review attempted such an analysis due to the potential unreliability of the authors (e.g. in anticipation of undiscovered research misconduct).

## Discussion

### Main findings

No set of inclusion criteria for studies within any evidentiary meta-synthesis includes a procedure for assessing the potential trustworthiness of authors. But, as 81 separate Cochrane reviews included a minimum of one paper from researchers who have undergone a catastrophic loss of trust (with 32 included specially in reviews), perhaps they should.

Some individual examples bear mention: in a review named “Interventions for preventing falls in older people living in the community [18]”, the authors decided to retain a retracted study in the final analysis, despite being alerted to its status by a reviewer. They replied to the reviewer’s comment that the retracted study was not a central contributor to the review’s conclusions and removing it could lead to data discrepancies in the extensive and stable review. Although the retracted study included in the review has been documented in the ‘Retraction Watch database [15], they denied doing a sensitivity analysis after the exclusion of the retracted study. Alternatively, there was a single case of a review author explicitly stating that a study from an author with multiple prior retractions should be deemed untrustworthy and questioning its inclusion.

When studies by authors with multiple retraction histories were excluded (42 cases) from the Cochrane reviews, it was generally for failure to meet the stated inclusion criteria of the review, not because of a stated lack of trustworthiness. This raises two intriguing possibilities. First, review authors may be excluding potentially unreliable papers with full knowledge that the paper is unreliable – generally, we can accept review authors are domain experts, and thus may well be aware of the most famous mass retraction cases in their specific fields – but may feel they have no justifiable method for stating their exclusion. Review authors may feel it is inappropriate somehow to explicitly state this as a reason for exclusion and may default to rejecting papers for inclusion using the fig leaf of the far less socially problematic failure to meet the relevant criteria.

It is likely that these papers, confined to a list of ‘all-time' dishonest authors, only represent a small percentage of a broader problem. As numerous systematic reviews reference Boldt, Fujii, and Asemi (all of whom are authors well known both in their fields and the popular press as serial fabricators), there are likely many more systematic reviews citing papers from less well-known, less prolific, but equally as dishonest authors. The extent of that problem is, in some part, defined by exactly how we choose to define a researcher with multiple retraction histories, where a formal recommendation might be made in order to treat a paper differently. While it feels outrageous to consider an unretracted paper by, say, Joachim Boldt (200 retractions at time of writing) as a trustworthy inclusion in a meta-result, it feels equally as outrageous to, say, reject for inclusion by definition a paper authored by a large workgroup where one single member has a single previous retraction.

While sensitivity analysis is a critical step in assessing the robustness of review findings, our findings show that only 19% of reviews conducted such an analysis after the exclusion of retracted studies or studies by authors with multiple retraction histories. This was, frankly, surprising. It represents a significant area for improvement in the systematic evaluation of aggregated research, and could also include an appreciation of the above: a responsible meta-analyst could attempt to re-run analyses discounting all papers where one or more authors has a retraction record, or after checking for bias/quality, LOO analysis, etc. via standard methods, report if these methods included or excluded studies from the same.

The paper published in Proceedings of the National Academy of Sciences, Richardson et al. [19] argues that contemporary scientific fraud is increasingly networked, coordinated, commericalzied and growing at an exponential rate, evading existing safeguards, thereby allowing such compromised research to permeate high-level evidence synthesis, including Cochrane reviews [19]. This suggests numerous other opportunities to harden meta-analytic results against the inclusion of unreliable studies. At a minimum, these include (a) the publication of brief, updated single figures (forest plots or otherwise within a meta-analysis if studies it relies on are later retracted or implicated by other retractions); (b) an editorial note stating places an interested reader might recalculate relevant results given the same; (c) a mandatory process of assessment for retraction or trustworthiness status if a meta-result is updated from an existing set of studies over time, as Cochrane reviews often are; (d) the broader construction and utilisation of tools which allow the author of meta-analyses to more easily achieve clarity on these issues without having to perform their own in-depth investigation of any given paper or researcher, and; (e) a move towards ‘living’ (i.e. updatable or modifiable) meta-analyses which explicitly allow for updates, changes, or modifications to be made by an editorial group. Given that the Cochrane Collaboration already provides all code and data used in the meta-analyses for readers for their reviews, these actions would be trivial to take and likely provide substantial benefits for medical science.

### Strengths and limitations

Our methodology was limited but robust. While much of the work was manual, the findings were supported by a range of careful secondary work, including using different search patterns and careful rechecking of the data by a second researcher to ensure validity. Within the confines of the research question, we are confident that we have identified every Cochrane review contaminated by the more notorious researchers who inhabit the upper echelons of the Retraction Watch database.

This study provides a preliminary exploration into the prevalence and implications of unreliable studies in Cochrane systematic reviews. However, a notable limitation is the absence of statistical analysis to directly measure the impact of these studies on the conclusions of the systematic reviews. Moreover, our focus exclusively on Cochrane reviews and studies authored by individuals with a history of 24 or more retractions may limit the generalizability of our findings. Given the context, it is almost certain that Cochrane reviews contain many more problematic papers from authors who are less notorious. Other, less robust reviews will also likely contain additional instances of fraud, given the extreme care that the Cochrane collaboration takes with their investigations.

Additionally, limiting our study to authors with 24 or more entries into the database might not capture the broader range of issues presented by other systematic reviews from authors with fewer retraction histories, which could still affect the reliability of the evidence. Future research should employ rigorous quantitative methods to more definitively assess the impact of studies by authors with multiple retraction histories. Expanding the scope to include a broader range of systematic reviews and evaluating the influence of studies from authors with varying levels of retraction records would further enhance our understanding of this critical issue. This may be solvable with a programmatic solution if there is the ability to access the Cochrane database directly.

### Implications of findings

Cochrane reviews are the gold standard for medical practice globally. The fact that there are numerous papers across more than a dozen reviews from authors who are well-known to have serious issues with their work is a worrying and problematic finding. There are almost certainly patients being harmed on a daily basis by the promulgation of unreliable research within systematic reviews. Empirical evidence has shown that studies at high risk of bias or with unreliable findings tend to overestimate treatment effects, which can mislead clinical decision-making [20, 21]. Furthermore, the inclusion of problematic or retracted studies in meta-analyses has been shown to influence pooled estimates and, in some cases, alter the overall conclusions of reviews, highlighting the importance of identifying and addressing such studies [22, 23]. Our paper certainly shines a light on a seriously underappreciated and under-researched topic.

In addition, the project shows the further impact of problematic research. Many, although not all, of the papers from these authors are of very low quality and are rated as such in their respective reviews. By including these studies in systematic reviews, the reviews increased their reach and impact, despite their low-quality ratings. This is a natural element of meta-scientific research, and indeed a key aim of meta-analyses, but it relies on a system of trust. We believe that this element of trust is unearned and, based on our results, it is imperative that reviewers begin taking into account potential misconduct in their reviews to ensure patient safety.

The findings also advocate for the incorporation of stricter methodologies to identify and exclude such potentially fraudulent studies authored by individuals with a history of retractions. And when these studies are involved, sensitivity analysis should be done and become a standard practice in systematic reviews, after excluding such studies. Conducting such analyses allows us to assess how the inclusion of these studies might influence review outcomes [24]. Finally, the research supports the establishment of protocols for routinely updating the systematic reviews when new retraction information becomes available. This approach can enhance the reliability of systematic reviews and maintain the integrity of evidence, ensuring clinicians are guided by high-quality research. This process could also, to an extent, be automated, with Cochrane reviewers containing retracted papers marked as such if any of the records used in the analyses are eventually retracted.

Finally, ensuring methodological rigor to exclude potentially fraudulent studies, performing regular sensitivity analyses, and routinely updating systematic reviews about new retraction information are not the responsibility of a single officer. It requires a collective commitment to integrity from everyone involved, including academic institutions, funding bodies, researchers, publishers, journals, editors, peer reviewers, and the wider clinical trial community, including clinicians [25].

How this relates to the general quantity and issue with fraud in systematic reviews not conducted by the Cochrane Collaboration is more problematic. In this paper, we have considered the most notable individuals who have been included in the highest-quality reviews that currently exist in the medical sphere. This is almost certain to represent the lowest possible lower bound of the rate of such unreliable research being included in systematic reviews. While Cochrane is already developing useful methods to prevent future issues such as this, most systematic reviews are conducted less robustly and likely will not add in substantial additional analyses to exclude potentially fraudulent studies and will never be updated in the ways discussed above.

Despite the low proportion observed (0.3%, 32 out of 9323) that included at least one study from authors with multiple retraction histories, this raises a question about the reliability of systematic reviews, as even rigorous reviews may include problematic research. There are innovative methods of excluding such studies in future reviews, such as the INSPECT-SR project, but these will not correct the existing enormous body of scientific research that currently drives medical care.

### Future research

Future work could build on the present findings by (1) excluding formally retracted studies from meta-analyses and comparing results to assess how such retractions influence pooled effect estimates and overall conclusions and (2) conducting sensitivity analyses that exclude studies authored by individuals with multiple retraction records to evaluate the robustness of meta-analytic findings and the potential impact of research by authors with a history of retractions. Alternative thresholds for defining highly retracted authors could also be explored to determine how different operational definitions influence conclusions. In addition, integrating retraction metadata directly into systematic review software would allow reviewers to more efficiently identify and flag retracted or potentially fraudulent studies, enhancing transparency and methodological rigor. Collectively, these approaches could improve the reliability and interpretability of aggregated evidence while providing guidance for responsible meta-analytic practice.

## Conclusions

This study highlights a critical and underappreciated vulnerability within evidence synthesis: the inclusion of potentially fraudulent studies authored by individuals with a history of retractions in Cochrane systematic reviews. The findings underscore the need for systematic reviewers and methodological bodies to incorporate tools and stricter guidelines that assess the trustworthiness of authors. These may include automated alerts when included studies are retracted, living updates to meta-analyses, and guidance for addressing studies from authors with significant retraction histories. Additionally, the mandatory use of sensitivity analyses should become standard practice when systematic reviews include such studies from authors with multiple retraction histories. While we acknowledge the complexities and ethical concerns in judging author credibility, the risk of perpetuating flawed or fraudulent evidence within high-impact clinical reviews demands careful scrutiny and transparent processes. As the number of retractions continues to grow and misconduct becomes increasingly visible, maintaining the integrity of systematic reviews must involve proactive strategies to detect and mitigate the influence of unreliable research.

## Data Availability

Dataset for investigating the prevalence of studies by retracted authors in Cochrane reviews, 2024 (https://docs.google.com/spreadsheets/d/1W-FVBdIEm740-qbF7mh122gYJyJH7ee-/edit?usp=sharing&ouid=103701781136960235054&rtpof=true&sd=true).

## Glossary

Cochrane Reviews: Systematic reviews produced by the Cochrane Collaboration, recognized for rigorous methodology and often used to inform clinical guidelines and healthcare policy.
Highly Retracted Authors: Researchers identified as having 24 or more retractions or corrections in the Retraction Watch Database at the time of data collection.
INSPECT-SR project: An initiative aimed at improving the quality of systematic reviews by developing tools to detect and address potentially fraudulent or unreliable studies.
Leave-One-Out (LOO) Analysis: A sensitivity analysis technique in which one study at a time is removed from the meta-analysis to assess its influence on the overall results.
Meta-analysis: A statistical method used in systematic reviews t combine data from multiple studies to produce a single pooled estimate of effect or outcome.
Meta-epidemiological Study: A study examining how characteristics of individual studies influence the outcome of systematic reviews and meta-analyses.
Retraction Watch Database: A publicly maintained database that tracks retracted scientific articles and maintains a list of authors with multiple retractions.
Scientometric Analysis: The quantitative study of publication patterns and metrics in scientific literature, such as citation counts and author productivity.
Sensitivity Analysis: A method used to test the robustness of review findings by re-running analyses after removing specific studies or changing assumptions.
